# Real-Time Robotic Presentation Skill Scoring Using Multi-Model Analysis and Fuzzy Delphi–Analytic Hierarchy Process

**DOI:** 10.3390/s23249619

**Published:** 2023-12-05

**Authors:** Rafeef Fauzi Najim Alshammari, Abdul Hadi Abd Rahman, Haslina Arshad, Osamah Shihab Albahri

**Affiliations:** 1 Center for Artificial Intelligence Technology, Faculty of Information Science & Technology, Universiti Kebangsaan Malaysia (UKM), Bangi 43600, Selangor, Malaysia; p103023@siswa.ukm.edu.my (R.F.N.A.); haslinarshad@ukm.edu.my (H.A.); 2 College of Science, University of Kerbala, Karbala 56001, Iraq; 3 Victorian Institute of Technology (VIT), Melbourne, VIC 3000, Australia; osamahsh89@gmail.com; 4 Computer Techniques Engineering Department, Mazaya University College, Nasiriyah 64001, Iraq

**Keywords:** presentation scoring, machine learning, fuzzy Delphi, AHP, evaluation, educational robot, human–robot interaction

## Abstract

Existing methods for scoring student presentations predominantly rely on computer-based implementations and do not incorporate a robotic multi-classification model. This limitation can result in potential misclassification issues as these approaches lack active feature learning capabilities due to fixed camera positions. Moreover, these scoring methods often solely focus on facial expressions and neglect other crucial factors, such as eye contact, hand gestures and body movements, thereby leading to potential biases or inaccuracies in scoring. To address these limitations, this study introduces Robotics-based Presentation Skill Scoring (RPSS), which employs a multi-model analysis. RPSS captures and analyses four key presentation parameters in real time, namely facial expressions, eye contact, hand gestures and body movements, and applies the fuzzy Delphi method for criteria selection and the analytic hierarchy process for weighting, thereby enabling decision makers or managers to assign varying weights to each criterion based on its relative importance. RPSS identifies five academic facial expressions and evaluates eye contact to achieve a comprehensive assessment and enhance its scoring accuracy. Specific sub-models are employed for each presentation parameter, namely EfficientNet for facial emotions, DeepEC for eye contact and an integrated Kalman and heuristic approach for hand and body movements. The scores are determined based on predefined rules. RPSS is implemented on a robot, and the results highlight its practical applicability. Each sub-model is rigorously evaluated offline and compared against benchmarks for selection. Real-world evaluations are also conducted by incorporating a novel active learning approach to improve performance by leveraging the robot’s mobility. In a comparative evaluation with human tutors, RPSS achieves a remarkable average agreement of 99%, showcasing its effectiveness in assessing students’ presentation skills.

## 1. Introduction

Technology has revolutionised the way we approach education, and its impact is particularly visible in the classroom. Today, technology is being used to improve teaching methods, enhance the learning experience of students and facilitate collaboration and communication between educators and students. Classroom technology refers to the wide range of digital tools and platforms that support classroom learning [1]. These tools include interactive whiteboards, digital projectors, tablets and online learning management systems [2], which help engage students in the learning process, provide them with instant feedback and help educators assess their students’ progress and adapt their teaching methods [2,3]. Robotics is another rapidly growing area of technology that is increasingly being used in education [4,5]. Robotics can be used to teach students important skills in STEM fields [6] and critical thinking and problem-solving skills [7,8]. Robotics also allows students to engage in hands-on learning experiences and build and programme robots to perform specific tasks [9,10].

Assessing students’ presentation skills is an important aspect of evaluating their communication and critical thinking abilities [11]. To effectively assess students’ presentation skills, it is essential to establish clear criteria that align with the learning objectives of their presentation. These criteria may include certain elements, such as organisation, delivery, use of visual aids and audience engagement [12]. In addition to observing the presentation itself, students should be given feedback on their performance, highlighting those areas where they performed well and where they need to improve [13]. Providing students with targeted feedback can help develop their presentation skills and shape them into confident and effective communicators [13]. Robotics can be effectively used in assessing students’ presentation skills and enhancing their learning experience. For example, educators can incorporate robotics into a presentation assignment to help their students improve their delivery and use of visual aids. Students can work in teams to build and programme robots that can assist with their presentation, such as a robot that can display images or interact with the audience. By incorporating robotics into presentation assignments, educators can help students develop their critical thinking and problem-solving skills whilst also providing them with a unique and engaging learning experience. This approach can also accustom students to using technology in their presentations, which is becoming increasingly important in the digital world.

A robotics-based objective scoring of presentation skills requires capturing all aspects of students’ performance with a focus on their face, body, and hand movements. Capturing these aspects necessitates the incorporation of reliable machine learning/deep learning models into other improvement algorithms [14]. Other important aspects, such as the real-time aspect and the use of different angles and positions for capturing student performance, also warrant consideration. Researchers have proposed various criteria for robotics-based presentation skills scoring, such as academic emotion recognition [15,16], eye contact [13,17], body movement [13], hand movement [13], audio [17], slides content [13] and duration [11]. Haider et al. (2020) developed an automatic scoring system for presentation skills, focusing on audio, posture, body language, idea structure and overall delivery, employing unsupervised data representation and machine learning techniques such as Gaussian process and support vector machines (SVM) [17]. Ochoa et al. (2018) introduced an assessment system, RAP, which utilised low-cost sensors for multimodal analysis, evaluating presentation skills based on posture, gaze, sound volume, filled pauses and slides, incorporating supervised deep learning and feedback reports [13]. Tun et al. (2023) presented a transfer learning framework for improving model performance in assessing oral presentations, focusing on feature extraction from audio, visual and text data and demonstrating the effectiveness of transfer learning [18]. However, studies on robotics-based presentation scoring approaches and systems have ignored four salient criteria, namely academic emotion, eye contact, body movement and hand movement. Specifically, no previous study has concurrently examined all of these non-verbal criteria, although some studies have considered at least three of these criteria. Moreover, eye contact, which is pivotal to evaluating presentation skills due to its indispensable role in audience engagement, has been conspicuously absent even when face recognition is involved in such scoring approaches and systems. Previous studies have also insufficiently utilised static and active sensing mechanisms. The latter refers to the actuation of robot movements, which serves to optimise the predictive accuracy of each individual non-verbal sub-criterion. As such, there is a palpable gap in the literature when it comes to the integrated assessment of these non-verbal criteria via robotic approaches that also employ active learning techniques for enhanced predictive capability. We aim to fill such gaps by proposing the multi-model robotics-based presentation skills scoring (RPSS) whilst jointly considering four important factors that are selected using fuzzy Delphi, namely facial expressions, eye contact, hand gestures and body movement, all of which are treated as aspects for emotion prediction. We create a separate machine or deep learning model for each of these criteria. We use the analytic hierarchy process (AHP) for weighting or prioritising the criteria used in scoring, and we apply robot control to improve emotion prediction performance by enabling active learning.

The rest of this article is organised as follows. Section 2 highlights the contributions of our work. Section 3 presents a literature survey. Section 4 discusses the research methodology. Section 5 presents the experimental results. Section 6 concludes the paper and offers suggestions for future work.

## 2. Contributions

Our article offers several contributions as follows:

1. To the best of our knowledge, this article is the first to propose a multi-model analysis approach for presentation scoring based on four criteria, namely facial expressions, eye contact, hand gesture and body movement. The proposed RPSS captures the sensing data by using an Intel real sensor D435 camera mounted on a turtle robot with a GUI interface for interaction.
2. RPSS identifies five academic facial expressions and eye contact to improve its scoring evaluation accuracy compared with other approaches that only rely on facial expressions without eye contact.
3. RPSS adopts fuzzy Delphi for criteria selection and incorporated AHP for weighting or prioritizing the criteria used in scoring. The outcome of Fuzzy Delphi-AHP is a weighting vector that assigns different weight for each criterion according to the system manager preference. The manager of RPSS defines the relative importance between the criteria and AHP to calculate the weights.
4. RPSS generates the final score by following predetermined rules that are formulated based on the selected criteria whilst considering the weights of each criterion according to the AHP-based estimation method.
5. RPSS maximises its prediction accuracy by using plausible actions from the robot (active learning) before providing its predictions to the AHP for calculating the score.
6. The study introduces the concept of active learning for robotic presentation scoring, where the robot actively adjusts its position to improve the quality of data recording, by leveraging active learning techniques. Active learning demonstrates the potential to enhance the prediction performance of presentation scoring systems.

## 3. Literature Review

The literature review is divided into two parts. The first part focuses on presentation scoring using artificial intelligence (AI) models, whilst the second part focuses on basic and learning-centred emotion classification.

### 3.1. Presentation Scoring

Few studies have explored presentation scoring using AI models. For instance, the authors in [17] designed an automatic scoring system for presentation delivery skills that uses a novel active data representation method to automatically rate segments of a video presentation. They conducted unsupervised data representation for video classification using low-level audio–visual descriptors and self-organising mapping and then used this representation to analyse the presenters’ performance characteristics, such as audio, posture and body language, idea structure and connection and overall presentation delivery. They built an automatic scoring system based on two top machine learning methods for regression, namely the Gaussian process and support vector machines (SVM). The authors in [11] designed a model for automatically detecting student behaviour to be used in assessing student presentations. They adopted a combination of computer-vision libraries and machine learning algorithms to produce their proposed model using video content. They also investigated human behaviours and their relations with personal modalities by using pattern recognition techniques. The authors in [13] proposed RAP (Spanish acronym standing for “automatic presentation feedback”), a system that offers automatic multimodal analysis and feedback on students’ oral presentation skills using low-cost sensors (Raspicamera). This system analyses students’ posture, gaze, sound volume, filled pauses and slides and utilises supervised deep learning approaches, such as a trained random forest model for eye contact and OpenPose C++ Library for skeleton detection. This system also sends an offline feedback report to the students along with a recording of their presentation. Tun et al. (2023) explored the use of a simple yet effective transfer learning framework for improving model performance in evaluating oral presentations in a target domain. They adopted a multimodal approach and extracted features from audio, visual and text modalities using specialised tools, such as COVAREP, OpenFace and Genism. They also compared their approach with various pre-trained models, including YAMNet, VGGish, VGG, MobileNet, BERT, LSTM and Stacked-LSTM, with and without the application of transfer learning to assess its impact on accuracy and robustness [18]. Their results highlighted the effectiveness of transfer learning in improving model performance.

We summarise these approaches in Table 1. None of these methods have used depth data as a complement to videos for scoring. Depth data can provide additional information about presenters’ body movements and distances from the camera, which can enhance the accuracy of scoring. Moreover, no previous study has combined face, eye contact, hand gestures and body movement in a single approach. Combining these multiple modalities can provide a highly comprehensive analysis of presentation skills and thus offer a more accurate scoring. As shown in the table, our work is distinguished from the existing works in the literature by jointly relying on depth and video sensing, facial emotion, eye contact, hand gestures, body movement and robots for presentation scoring.

### 3.2. Basic and Learning-Centred Emotion Classification

Emotion recognition is a crucial functionality for presentation scoring. This process involves identifying and interpreting emotions expressed by humans via their facial expressions, speech and body language. Facial expression plays a vital role in emotion recognition. Learning-centred emotions are a specific subset of emotions that are experienced during the learning process, such as confusion, boredom, engagement and frustration, and recognising these emotions can improve students’ learning experience by offering personalised feedback and interventions. The approaches proposed in the literature can be categorised into learning-centred emotion-aware approaches and basic emotion-based approaches.

For the first category, the authors in [19] investigated the automated detection of uncertainty based on facial expressions in a learning context by using the facial action coding system. They collected and annotated facial expression data before the pre-processing and feature extraction stage. Afterwards, they used Gabor wavelets to extract features from images and adopted SVM for the data classification. The authors in [20] developed a recognition model for detecting academic confusion in online learning based on facial expressions. Their model involves three processes, namely confusion-inducing experiments, image pre-processing and comparison of recognition methods. They utilised a combination of machine and deep learning methods (i.e., histogram of oriented gradient (HOG), local binary patterns (LBP), SVM and convolutional neural network (CNN)) to develop four new approaches, namely HOG-SVM, LBP-SVM, CNN and CNN-SVM.

The authors in [21] designed a student engagement evaluation system that uses a laptop web-camera in real-time lecture sessions. This system combines information on the eyes and head with facial expressions to produce a concentration state in e-learning scenarios. This system was developed using machine and deep learning techniques, specifically the Haar cascade algorithm and CNN. The authors in [22] developed a robot that employs a recognition model for analysing children’s fatigue state during learning via multi-cue fusion. This model captures the facial features of children, specifically their mouths and eyes, and relieves their fatigue and improves their learning efficiency via multi-channel interactions, such as voice, image, and sound.

The second category can be divided into two sub-parts, one of which leverages body movement, whilst the other ignores body movement. For the first sub-part, the authors in [23] proposed a method for the automatic recognition of affect that leverages body and facial expressions. They used hierarchical multi-label annotations and multi-stage losses for deep learning models. They trained their models jointly and separately and designed computational models for both individual modalities and whole-body emotions. Meanwhile, the second sub-part can be further subdivided into two, the first of which ignores eye contact.

The authors in [24] proposed a learner’s emotion recognition model in the online learning scenario and constructed an intelligent education system called Smart-E based on this model. This model utilises deep neural networks and SVM to recognise and analyse learners’ facial images in real time to understand their emotional states. The direction and size of learners’ emotional vectors were then calculated based on the expression classification and blink frequency. The authors in [25] proposed a feature sparseness-based regularisation that learns deep features and shows improved generalisation ability for face recognition. They also proposed a deep metric learning framework to optimise the regularisation of the recognition network, which is integrated into the SoftMax loss function. The authors in [26] proposed an automatic facial expression recognition system that combines a new video pre-processing method with CNNs, namely AlexNet, GoogleNet and ResNet. This new pre-processing method was designed based on the idea that each human emotion is dynamic; that is, facial changes are treated as essential features. The authors in [27] proposed the Korean video dataset for emotion recognition that is collected from Korean movies. This dataset is useful for studying the facial emotions of Eastern people, particularly Koreans, in close-to-real-world conditions. They also developed a semi-automatic video emotion-labelling tool to annotate facial expressions in video clips. During the experiments, they applied baseline deep learning models, including VGG16 and multi-layer perceptron, to determine the quality of the proposed dataset. The authors in [28] proposed an automatic face emotions recognition model that was embodied in a smart doll in a learning environment. This model was designed based on the capabilities of an Eyes of Things device and the deep learning techniques for a real-life facial informatics application. The authors in [29] designed a deep CNN architecture pre-trained as a stacked convolutional autoencoder (SCAE) to achieve emotion recognition in unconstrained environments using the Nao robot. This model combines convolutional and fully connected layers and uses SCAE that was trained in a greedy layer-wise unsupervised fashion (Gradual-GLW) to encode facial expression images as facial pose and illumination-invariant reconstructions.

The approaches falling under the second sub-part consider eye contact. The authors in [30] proposed a new eye contact detection method that robustly detects eye contact by using cameras without specialised eye tracking equipment poses. This method was trained on a natural group interactions dataset and has enhanced over a head pose-based baseline. The authors in [31] developed an automatic eye contact detection model based on a deep neural network model from video images. The model used the Deep Eye Contact (DeepEC) model. They also collected a new dataset from children with autism by using a pair of commercially available camera glasses to examine the non-verbal communication behaviours present in typical children’s development.

Upon reviewing Table 2, we observe that none of the existing algorithms has jointly considered all factors, including face, eye contact and body and hand movements, in predicting basic or learning-centred emotions. We also observe that different aspects of development are considered when predicting emotions. Whilst some researchers have used mathematical models for feature extraction, such as local binary pattern, Gabor filter, PHOG and LGBP, they used dimensionality reduction approaches, such as PCA and LDA, before presenting the features to the classifier [15]. Some approaches have also used pre-trained models, such as Inception v3, VGG16 and ResNet [15,25], with or without transfer learning. Nevertheless, the different aspects that are considered in the prediction require extracting more than one type of feature and fusing them, and different models are considered for future fusion.

## 4. Methodology

This section discusses the research methodology. We initially discuss our criteria collection and selection, followed by our criteria weighting and data collection procedures. Afterwards, we describe our skeleton, face and eye identification and learning-centred emotions classification, which is followed by hand gesture detection and body movement analysis. We also discuss our active learning algorithm for improving scoring performance and conduct a case study to understand the experiences of users with our approach and solicit their feedback.

### 4.1. RPSS

**Figure 1:**
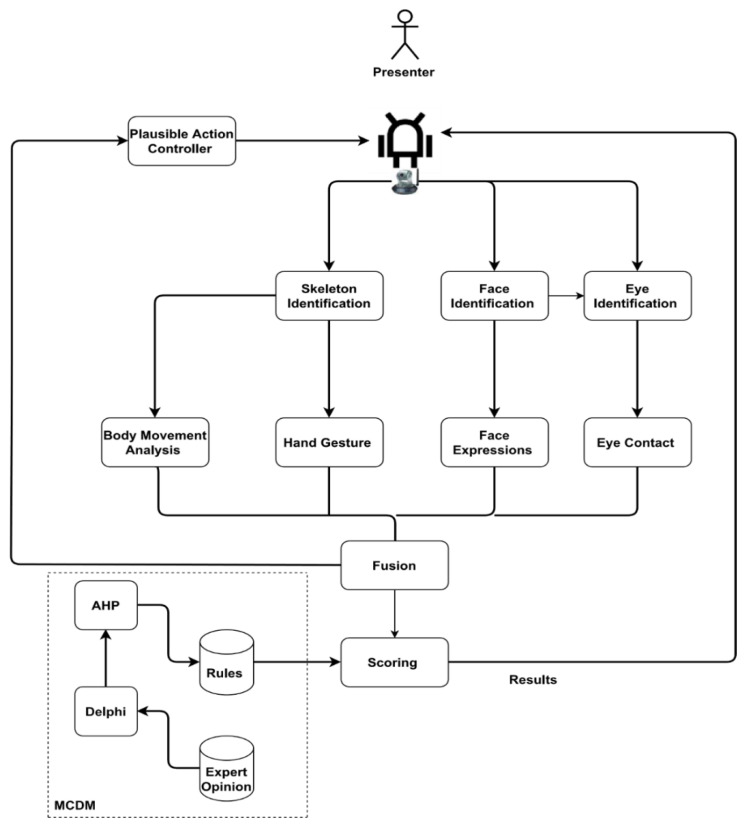
Developed scoring approach for our multi-model analysis and RPSS.

Figure 1 illustrates our proposed multi-model analysis and robotic decision-making approach. The presenter delivers his presentation in front of a video camera installed on the robot, and a video is captured and analysed in real time. Three types of features are extracted, namely skeleton, face and eye identification features. Each of these features is then sent to different blocks corresponding to eye contact detection, facial expressions identification, hand gesture detection and body-movement analysis. These features are then provided to the fusion unit, whose output is fed to the scoring unit. The scoring receives external knowledge from the rules provided based on the weights calculated by fuzzy Delphi. Delphi is a consensus-based decision-making method that involves a panel of experts providing their opinions on a given topic. Accordingly, RPSS uses Delphi for criteria selection. The experts in this case may include educators, public speakers or other professionals with experience in the field. These experts are asked to provide their opinions on which criteria are most important for evaluating presentation performance. These opinions are then collected and analysed to determine the most important criteria, which are then used by the scoring unit to assign scores to the presenters’ behaviour. The experts’ opinions used for criteria selection are collected by conducting interviews with experts, including psychological experts. The opinions shared by these experts are critical in ensuring that the selected criteria for evaluation are relevant and comprehensive. By including experts with different backgrounds and perspectives, the Delphi method ensures that the selected criteria are comprehensive and reflective of the best practices and standards in the field.

The pseudocode is presented in Algorithm 1. In the provided pseudocode, the real-time video stream captured by the robot’s camera, denoted as videoStream, and a collection of criteria from experts, referred to as expertOpinions, serve as the primary inputs. Additionally, scoringCriteria, as determined by the Delphi method, and AHPWeights, derived from the AHP method, are also utilised as inputs. The ultimate goal of the algorithm is to produce an output called scoringResult, which classifies the presenter into one of four groups: A, B, C or D. Initially, features related to the skeleton of the presenter are extracted from the video stream. Subsequently, features associated with the presenter’s face are extracted, followed by features related to the eyes. Once these features are extracted, they are further processed to detect eye contact, identify facial expressions, and recognise hand gestures and body movements. After these analyses, a fusion process is employed to consolidate the detected eye contact, identified facial expressions, hand gestures, and body movements into a unified data structure named fusedData. With the fused data in hand, a scoring unit is then employed, utilising both the fused data and the predetermined scoring criteria. This scoring unit generates a score which represents the performance of the presenter. In the final step, a rule-based classification method is used, taking into account the score and the AHP weights, to categorise the presenter’s performance into one of the predefined groups: A, B, C or D. Once this classification is determined, the scoring result is returned as the final output of the algorithm.
**Algorithm 1:** Pseudocode of our proposed RPSS**Input:** *  (1) videoStream—real-time video stream from the robot’s camera.* *  (2) expertOpinions—collection of criteria from experts.* *  (3) scoringCriteria—as found by Delphi method.* *  (4) AHPWeights—as found by AHP method.* **Output:** *  scoringResult—group A, B, C, or D.* 1: **Start algorithm**: *2: skeletonFeatures = ExtractSkeletonFeatures(videoStream)* *3: faceFeatures = ExtractFaceFeatures(videoStream)* *4: eyeFeatures = ExtractEyeFeatures(videoStream)* *5: eyeContact = EyeContactDetection(eyeFeatures)* *6: facialExpressions = FacialExpressionsIdentification(faceFeatures)* *7: handGestures = HandGestureDetection(skeletonFeatures)* *8: bodyMovements = BodyMovementAnalysis(skeletonFeatures)* *9: fusedData = Fusion(eyeContact, facialExpressions, handGestures, bodyMovements)* *10: score = ScoringUnit(fusedData, scoringCriteria)* *11: scoringResult = RuleBasedClassification(score,AHPWeights)* *12: Return scoringResult* **13: End algorithm.**

#### 4.1.1. Criteria Collection and Selection

This study adopted the Fuzzy Delphi method, which is one of the most effective MCDM approaches, to screen the evaluation factors of the automated multi-classification evaluation model. Helmer and Dalkey proposed this approach in the 1950s as an expansion of the Delphi approach. It has been chosen for this study due to the inherent imprecision, subjectivity, and ambiguity in expert opinions, which cannot be precisely quantified into clear data for real-world systems [32]. The fuzzy set theory was recommended to address this issue, making it an effective method for decision-making with a limited sample size of experts. The fuzzy Delphi method can also be applied to solve the fuzziness of a common understanding of expert opinions on a group decision [33].

The Gaussian Fuzzy Delphi is based on fuzzy set theory. If U is universal, then the fuzzy set of U is defined as described by [33]. The Gaussian membership function is mathematically expressed in Equation (1), and a Gaussian number is further elaborated with the help of an alpha value in Equation (2) [33]. These Gaussian Fuzzy numbers are then returned into interval arithmetic, generating the interval for fuzzy Delphi.

(1) $${µ}_{ɑ}=Gaussian(\chi ,µ,\sigma )=exp(\frac{-{\left(\chi -\mu \right)}^{2}}{\sigma^{2}})$$

A Gaussian number is expressed with the help of an alpha value. In the process, the lower and upper values of the fuzzy number can be taken as

(2) $$\chi =\left\{\begin{array}{c} \mu_{A}-\sqrt{\ln\left(\frac{1}{a^{\sigma^{2}}A}\right)}\;\mathrm{if}\;\chi <{µ}_{A}\;\\ \mu_{A}+\sqrt{\ln\left(\frac{1}{a^{\sigma^{2}}A}\right)}\;\mathrm{if}\;\chi \underline{>}\;{µ}_{A} \end{array}\right.$$

By returning Gaussian fuzzy numbers into the interval arithmetic, we generate an interval for the Fuzzy Delphi. This method can be divided into the following stages:

Stage 1: Identifying the yardstick and criteria for the research

When using the Fuzzy Delphi method, a research questionnaire should be developed for weighting the research criteria. The questionnaire items can be designed based on literature reviews, pilot studies and prior research experiences. Scholars agree that the items and content elements of a study should be constructed based on a review of the related literature [34].

An extensive review of the literature is necessary to specify the possible yardstick and criteria. Accordingly, we conduct a survey on various factors that have been used in the scoring literature. We identify nine factors, as summarised in Table 3, and use them to create the Fuzzy Delphi survey. Some sample questions are shown in Table 4, and the definitions of the selected factors are presented in Table 5. The setting of the Gaussian set is presented in Table 6.

We distribute a seven-point questionnaire (Table 6) amongst the experts to solicit their input [34]. We also design a questionnaire containing the students’ evaluation factors to solicit the preferences and opinions of the experts.

Stage 2: Collecting expert judgements and opinions via group decisions.

After clarifying the appropriate criteria, we invite a set of experts to measure the importance of these criteria by using linguistic variables, as shown in Table 6. A total of 20 experts in the field of education (including psychologists) and computer science have responded to our survey. We also interview these experts to gain a comprehensive understanding of their responses. The inputs of each expert are converted into fuzzy numbers as shown in Equations (3) and (4) [33,34]:

(3) $$Z_{ij}=\left(\mu_{ij},\sigma_{ij}\right)\;for\;i=1,..,n\;and\;j=1,..,m$$

where

n denotes the number of experts;
m denotes the number of factors.

We represent each $Z_{ij}$ by $\left[\mu_{ij}-\sqrt{\ln\left(\frac{1}{a^{\sigma^{2}}ij}\right)},\mu_{ij}+\sqrt{\ln\left(\frac{1}{a^{\sigma^{2}}ij}\right)}\right]$ and perform averaging as follows:

(4) $$\bar{Z_{j}}=[\frac{1}{n}\left(\sum_{i}^{N}=1\mu_{ij}-\sqrt{\ln\left(\frac{1}{a^{\sigma^{2}}ij}\right)}\right),\frac{1}{n}\left(\sum_{i}^{N}=1\mu_{ij}+\sqrt{\ln\left(\frac{1}{a^{\sigma^{2}}ij}\right)}\right)]$$

The fuzzy weights of factors $\bar{P_{j}}$ are given by $\bar{P_{j}}=\bar{Z_{j}}$.

Stage 3: Specifying the criteria

The defuzzification value of each factor $P_{j}$= defuzzification $\left(\bar{P_{j}}\right)$ is then calculated and compared with the threshold γ, which is 0.5. The factor is accepted in the case $P_{j}>\gamma$ and is rejected otherwise. The overall weights of the Gaussian fuzzy numbers for each factor are presented in Table 7. The values are all higher than 0.5, which means that all of these factors are important and need to be selected for presentation scoring evaluation. We select facial expression, eye contact, hand gesture and movement and body posture and movement because they are classified as non-verbal emotion factors that are within the scope of this work.

Results of the Fuzzy Delphi survey indicate that facial expression, eye contact and hand and body movement are all important due to having weights above 0.5. Therefore, we select these factors for further analysis.

Stage 4: Consistency checks

The results of Fuzzy Delphi are then validated based on the inconsistency index (I.I) and the random number of inconsistency index (R.I.I). The value of I.I. for each comparison matrix is obtained using the following [37]:

$$I.I=\frac{\lambda_{max}-n}{n-1}$$

Afterwards, the value of I.I. is calculated by random numbers (R.I.I.). The values of R.I.I. for (1 to 9) dimensional matrices are (0, 0, 0.58, 0.9, 1.12, 1.24, 1.32, 1.41, 1.45). For each n × n square matrix, the result of I.I. divided by R.I.I. is called I.R. or consistency [37]:

(5) $$I.R=\frac{I.I}{R.I.I}$$

The closer the I.R. is to zero, the more consistent the result. If I.R. is more than 0.1, then the decision should be reviewed. The IR in this study is 0.04, thereby validating the consistency of our results.

#### 4.1.2. Criteria Weighting

We adopt AHP for criteria weighting. We ask 18 experts to compare each factor with others in terms of importance [37,38]. These experts are different from those who have participated in the Fuzzy Delphi survey to avoid bias, to cross-validate our results and to prevent one method’s results from influencing those of another [39].

For each pair of factors, we ask the experts to indicate whether one factor is extremely more important, more important, equally important, less important or extremely less important than the other factor. Afterwards, we build an AHP matrix that quantifies the experts’ opinions following the AHP approaches proposed in [39]. This matrix has a 4×4 dimension because we have four factors, namely eye contact, facial emotion, hand gesture and body movement. The weight of the four criteria based on AHP is presented in Table 8.

Eye contact obtains the highest weight of 0.47, followed by facial expression (0.28), hand movement (0.14) and body movement (0.09). The validation score is 0.08.

#### 4.1.3. Data Collection

The dataset is made up of 18,150 frames of 88 video snippets captured from 22 participants consisting of 12 females and 10 males from different races, namely Malay, Chinese, Indian, Bangladeshi and Arab. Based on the literature, a comparison of previous studies was created, categorising them into three ranges: studies with under 20 subjects, studies with 20–30 subjects and studies with more than 30 subjects. The analysis revealed that the majority of studies fell within the range of 20–30 subjects. Furthermore, in our study, we ensured diversity among the participants by including individuals from various nationalities, ages, and genders. The deliberate gender balance was maintained to address the possibility of gender-specific variations in emotion expression during the presentation sessions. The related statistics are presented in Table 9 and Table 10. We ask each participant to deliver a three-to-five-minute presentation in front of The TurtleBot robot equipped with a Microsoft Intel real sense D453 camera and a Jetson nano kit board showing an interface with a camera and motors. The camera captures the RGB and in-depth information for each presentation. Each participant delivers his/her presentation four times using a uniform slide content, and each of these presentations corresponds to different scoring levels in a lab environment. This is justified by the focus on other metrics, which correspond to the varying scoring levels across the four instances. The scoring levels are obtained from different percentages of eye contact, body movement, hand gestures and facial expressions. The distance between the presenter and the camera in our study was 0.95 m. This choice of distance aligns with the recommendations found in the literature as the optimal distance [40]. Basically, the input video captured in this study encompassed the facial and upper body regions of the presenters. This specific framing of the captured video content was chosen to focus on the presentation-relevant aspects while providing a holistic view of the presenter’s non-verbal communication cues and expressions. In this study, a frame selection approach was employed, with one frame being chosen from every 15 extracted frames, as the redundancy in the full set was often observed. This method was used to optimise computational efficiency while preserving essential visual data for analysis.

#### 4.1.4. Skeleton Identification

**Figure 2:**
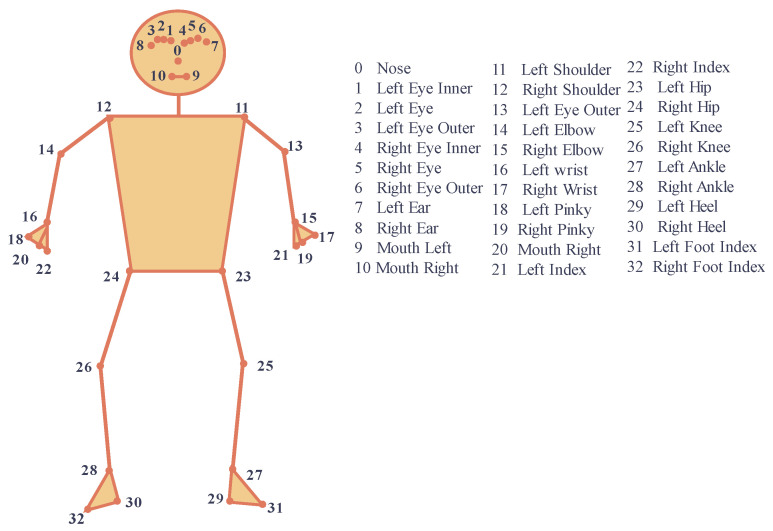
Joints generated from MediaPipe BlazePose.

For skeleton tracking, we use BlazePose [41], a lightweight CNN architecture for human pose estimation that is tailored for real-time inference on mobile devices. During inference, the network produces 33 body key points for a single person, as shown in Figure 2, making it suitable for real-time tracking.

#### 4.1.5. Face Identification

**Figure 3:**
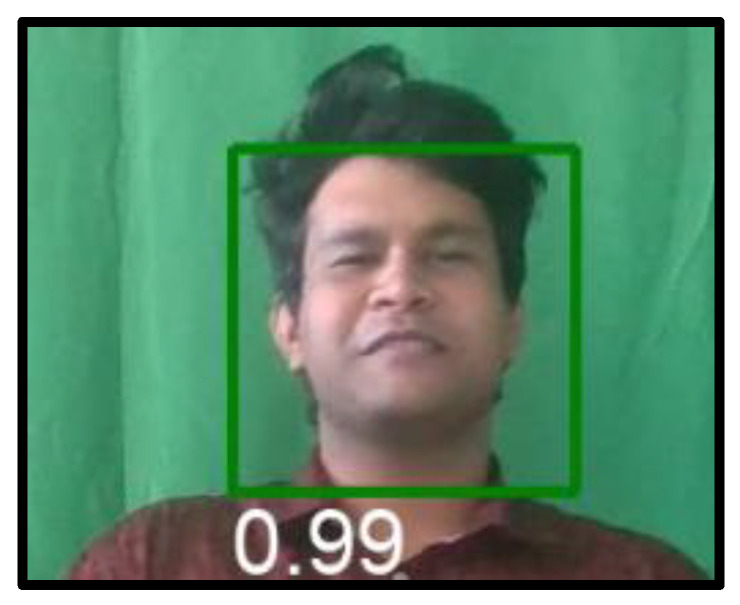
Face detection applied to one video from our dataset.

For face detection, we use the BlazeFace algorithm [42] from MediaPipe, which represents a machine learning model developed by Google, to rapidly detect key points from faces. This model is capable of predicting axis-aligned face rectangles and producing six facial keypoint coordinates (for eye centres, ear tragions, mouth centre and nose tip) that allow us to estimate face rotation (roll angle). From a design perspective, this algorithm enlarges the receptive size. For feature extraction, we use an extractor that takes an RGB input of 128 × 128 pixels and consists of a 2D convolution followed by 5 single BlazeBlocks and 6 double BlazeBlocks. For the anchor scheme, we predict a set of regression (and possibly classification) parameters, such as centre offset and dimension adjustments, for each anchor and use them to adjust the predefined anchor position into a tight bounding rectangle. As a post-processing step, to reduce the fluctuations of the bounding box amongst different frames, we replace the suppression algorithm with a blending strategy that estimates the regression parameters of a bounding box as a weighted mean amongst the overlapping predictions. Figure 3 provides one image from our built dataset with the bounding box of face identification with an associated confidence level of 99%.

Algorithm 2, “Extract Faces”, takes videos as input and outputs an array of frames, an array of extracted faces and the corresponding face coordinates for each frame. The algorithm starts by iterating through each input video and then extracts each frame from the video using the “extractFrames” function. For each extracted frame, the algorithm calls the “extractFaceCords” function to determine the coordinates of the face in the image. The coordinates are then stored in the “cords” variable. Afterwards, the algorithm crops the face image from the frame using the face coordinates and the “cutImage” function. The cropped face image is then appended to the “facesArray”. After iterating through all frames in the current video, the algorithm proceeds to the next input video and repeats the above procedure until all videos are processed. After processing all videos, the algorithm outputs three variables, namely the “framesArray”, which contains all the extracted frames; the “facesArray”, which contains all the extracted faces; and the corresponding “cords” for each face in each frame.
**Algorithm 2:** Pseudocode of extracting faces from videos**Input:** *  (1) Videos: The original videos.* **Output:** *  framesArray.* *  facesArray.* *  cords: face coordinates in image* 1: **start algorithm** *2:**for** each video in videos **do*** *3:   framesArray = extractFrames(video)* *4: **  for** each frame in framesArray **do*** *5:     cords = extractFaceCords(frame)* *6:    face = cutImage([cords])* *7:    facesArray = append(facesArray,face)* *8: **  end for*** ***9: end for*** **10:end algorithm**

#### 4.1.6. Eye Contact

To detect eye contact, we use the same model proposed by [31], which uses ResNet50. This model, recognised as DeepEC, employs deep CNN with a ResNet 50 backbone architecture as a classifier model. The model inputs cropped face regions that are resized to 224 × 224 pixels. The model also applies a two-stage training process to support task transfer learning. In the first stage, three public datasets are trained to learn the relationship between head pose and eye gaze direction. The model is trained to regress the 3D gaze direction based on the MPIIGaze [43] and EYEDIAP [44] datasets and the 3D head pose based on the SynHead [45] dataset with an L2 regression loss. Convergence is reached at <6° mean absolute error on gaze angle and head pose. The model is then finetuned using the training dataset to learn the condition of eye contact and to capture the details of facial appearance.

The parameters are fine-tuned across the last two blocks of ResNet layers using cross-entropy loss with a re-weighting factor of 0.1, which is multiplied by the loss of the over-represented class in order to compensate for the class imbalance (eye contact presence vs. absence ratio) in the used dataset.

Algorithm 3 has two inputs, namely the video path (videoPath), which identifies the location of the video to be analysed, and the model weight path (modelWeight), where the weights of the pre-trained DeepEC model are stored. This algorithm starts by initialising three lists, namely y (stores a binary result (0 or 1) indicating eye contact for each frame), confidence (stores the confidence of the eye contact prediction for each frame) and k (stores confidence values greater than 0.5). The pre-trained model weights are then loaded using the modelStatic function. A set of threshold values for confidence, total confidence and total score are established to determine the presence of eye contact. The algorithm loops through the frames of the video and uses a face detection model to locate the face in each frame and crop the surrounding image. The cropped face is then resized to a 224 × 224 pixel image and passed to the DeepEC model, which produces an output with calculated confidence. If such confidence exceeds the threshold, then y is updated as 1 (indicating eye contact); otherwise, y is updated as 0 (indicating no eye contact). If the confidence exceeds 0.5, then the confidence value is added to k. After all frames have been processed, the total confidence and total score are computed by taking the size ratio of k to y and the average of k, respectively. If both the total score and total confidence exceed their respective thresholds, then finalResult is set to 1 (indicating the presence of eye contact in the video); otherwise, finalResult is set to 0 (indicating the absence of eye contact in the video). finalResult is then returned, marking the end of the algorithm. In other words, for decision making in Algorithm 3, a confidence score for eye contact is obtained from the model’s output. This score is then compared to predefined thresholds to categorise the frame as either having eye contact or not. Aggregated statistics are calculated at the end of the video stream, including the ratio of frames exceeding a certain confidence level and the average confidence score across all frames. The final decision is made by comparing these aggregate metrics to their respective thresholds. If both metrics surpass the predetermined limits, eye contact is considered to have been present in the video; otherwise, it is considered absent. Thus, the final outcome is a product of both frame-level and aggregate-level evaluations.
**Algorithm 3:** Detect eye contact using Deep EC model**Input:** *  (1) videoPath: Path where the video is stored* *  (2) modelWeight: Path where weights of DeepEC model is stored* **Output:** *  (1) finalResult: it will be 1 if video with eye contact or 0 if not*  1: **start algorithm**  2: *y* = list stores 0 or 1 for each frame  3: *confidence* = *list stores confidence of eye contact for each frame*
  4: k = *list stores confidence then more 0.5*  5: *model = modelStatic(modelWeight)*  ▷ load model weights   6: *confidenceThresh = 0.9*  7: *TotalConfThresh = 0.75*
  8: *TotalScoreThresh = 0.85*  9: **while** videoCapture is opened **do**  10: *frame ← readFrame*  11: *boundedBox ← FaceDetectionModel(frame)*
  12: *face ← faceCrop(boundedBox)*  13: *image ← face.resize(224,224)*
  14: *output ← DeepECModel(image)*  15: *confidence ← confidence(output)*  16: **if**
*confidence* ≥ *confidenceThresh* **then**
  17: *    y ← 1*  18: **else**  19: *    y ← 0*
  20: **   end if**  21: **if** *confidence* > 0.5 **then**
  22: *    k ← confidence*  23: **end if**
  24: **end while**  25: *totalConf* ← size(k)/size(y)   26: *totalScore* ← average(k)  27: **if** *totalScore* > *TotalScoreThresh ∧ totalConf > TotalConfThresh* **then**
  28:  *finalResult ← 1*  29: **else**  30:   *finalResult ← 0*  31: end **if**  32: **return** *finalResult*  33: **end algorithm**

#### 4.1.7. Learning-Centred Emotions Classification

We develop a scoring approach that can classify facial expressions into five categories, namely boredom, engagement, confusion, frustration and delight, which are important in assessing student behaviour during presentations.

We use deep learning techniques, namely Xception, ResNet, MobilNet and EfficientNet to train the emotions classification models, as it will be covered in detail in the Experimental works and Analysis section. Specifically, we train these models on the Daisee dataset, which contains a large collection of video snippets captured from 112 users. This dataset contains four of the five facial expression categories of interest, namely boredom, engagement, confusion and frustration. The models are initially trained on this dataset to learn how to classify these four facial expressions.

However, given that the fifth facial expression category, delight, is not included in the dataset, we fine-tune the models on a custom dataset containing delight expressions to introduce this missing category. The processing sub-stages include data preparation and model training, which are further discussed as follows.

- Data Preparation

Algorithm 4, “Link Faces with Labels”, takes in cropped face images and their corresponding labels as input and then outputs a numpy array of linked face and label pairs. The algorithm begins by iterating through each face and label pair using the “zip” function. For each pair, the face image and label are converted into a numpy array using the “toNumpy” function. Afterwards, we call the “link” function using the face and label as inputs. This function outputs a numpy array containing the linked face and label, which are later appended to the “numpyFaces&labels” array using the “append” function. After iterating through all face and label pairs, the algorithm outputs the final “numpyFaces&labels” array containing all the linked face and label pairs.
**Algorithm 4:** Pseudocode of link faces with labels**Input:** *  (1) faces: images cropped to show only the face of the participant.* *  (2) labels: dataframe of daisee dataset.* **Output:** *   numpyFaces&labels.* *   face&label: each face image with it's corrosponding label.* 1:**start algorithm** *2: **for** each faces,label in zip(faces,label) **do*** *3:    face = toNumpy(face)* *4:    label = toNumpy(label)* *5:    face&label = toNumpy(link(face,label))* *6:    numpyFaces&labels = append(numpyFaces&labels,face&label)* *7: **end for*** 8: **End Algorithm**

B. Model Training

The Multi-Task Learning algorithm takes in the numpy array of linked face and label pairs, a pre-trained model, and pre-trained weights as input and outputs a trained model that can classify facial expressions into five categories.

The algorithm begins by loading the pre-trained model and weights using the “loadModel” function and then appends a fully connected layer to the model using the “append” function. Afterwards, for each of the five facial expression categories, the algorithm appends a corresponding output layer to the model, with the loss function set to “sparseCategoricalCrossentropy”.

Sparse categorical cross-entropy is a loss function commonly used in multi-class classification problems. This function is often applied when the true labels are integers rather than one-hot encoded vectors. The loss function is formulated as follows [46]:

(6) $$Loss=-\sum_{i=1}^{outputSize}y_{i}log(\hat{y_{i}})$$

In the context of facial expression recognition, one-hot encoding is used to represent the target labels for a multi-class classification problem, where each expression category is represented by a unique binary vector. One-shot learning is used in a scenario where only a few examples of each expression are available for training, and the goal is to learn a similarity metric between expressions.

After adding the output layers, the model is trained on the linked face and label pairs using the “fit” function with the “numpyFaces&labels” array as input data. The trained model is then outputted by the algorithm.

#### 4.1.8. Hand Gesture Detection

Hand gesture detection involves calculating the angle among three points (shoulder, elbow and wrist) for both the left and right hands and then comparing the difference between the angles in the current and previous frames. The frames are classified as positive or negative for hand movement, and groups of frames are classified based on the prevailing category. We detect the hand movements in the presentation to contribute to the evaluation of the presenter as follows:

Firstly, we calculate the angle amongst three of the seven available points (shoulder, elbow and wrist). For the right and left hands, we perform the following:

- Calculate the difference in Xs between the wrist and elbow points.
- Calculate the difference in Ys between the wrist and elbow points.
- Calculate arctan2 for previous values.
- Repeat the above steps for the shoulder and elbow points.

Secondly, we calculate the difference between the angles in the current and previous frames if the result is greater than a threshold when hand movement is detected. After classifying each frame, we divide all frames into groups and then classify each group according to the prevailing category.

Thirdly, we calculate the ratio of the number of groups classified as positive and then multiply the result by a certain percentage.

#### 4.1.9. Body Movement Analysis

We design a method that tracks three joint points (nose, left shoulder and right shoulder) and checks for any changes in their position and distance amongst frames. If a change is detected, then the frame is classified as containing body movement. The left and right shoulder points are only updated when a frame is classified as having body movement. The frames are then grouped, and the ratio of groups containing body movement is calculated and combined with a score for hand movement to produce the final rating for the video. The flag of body movement is set to 1 using Equation (7).

(7) $$bodyMovement=\left\{\begin{array}{c} 1\;if\left(x_{l,t}>x_{nose,t-1}\right)\;or\;\left(x_{r,t}<x_{nose,t-1}\right)\\ 0\;\;\;\;otherwise \end{array}\right.$$

where

$x_{l,t}$ denotes the x-coordinate of the left shoulder at moment t;
$x_{r,t}$ denotes the x-coordinate of the right shoulder at moment t;
$x_{nose,t-1}$ denotes the x-coordinate of the nose at moment t.

**Figure 4:**
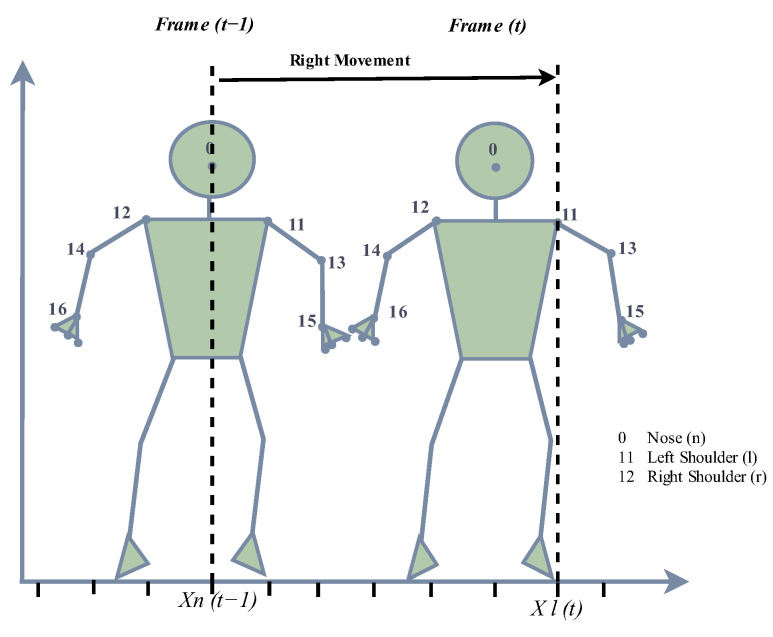
The detection of body movement, showing the right movement when the x-coordinate of the left shoulder at time (t) is higher than the x-coordinate of the nose at time (t − 1).

**Figure 5:**
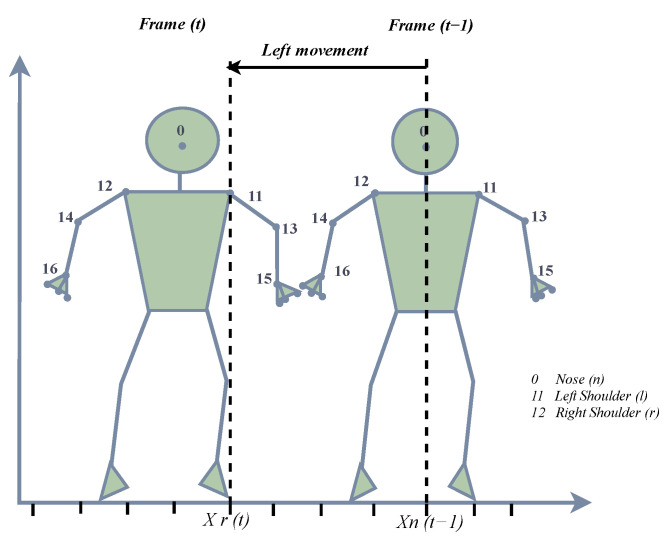
The detection of body movement, showing the left movement when the x-coordinate of the right shoulder at time (t) is lower than the x-coordinate of the nose at time (t − 1).

We track the body joints by applying hybrid techniques that use both the measurements from MediaPipe and the Kalman filter method. Hence, the body movement detection is designed to capture lateral shifts along the horizontal *x*-axis by comparing the x-coordinates of the left and right shoulders with that of the nose, as shown in Figure 4 and Figure 5. The robot’s movement, on the other hand, occurs along the longitudinal axis to maintain an optimal position relative to the presenter.

Because these movements are orthogonal and occur along different axes, the robot’s longitudinal movements do not interfere with the algorithm’s ability to accurately detect the lateral body movements of the presenter. Each is detected and processed in its own right without confounding the other.

#### 4.1.10. Rule-Based Scoring Model

The rule-based scoring approach assesses the performance of students based on their non-verbal emotions, which are categorised into four main scales or levels that define the range of emotions observed during the session:

1. Group A: Students in this group exhibit predominantly positive emotions, maintain consistent eye contact and frequently utilise hand and body gestures throughout the session.
2. Group B: Students in this group display a mix of positive and negative emotions, maintain moderate eye contact and make regular use of hand and body gestures.
3. Group C: Students in this group demonstrate a high prevalence of negative emotions, varying levels of eye contact and occasional use of hand and body movements.
4. Group D: Students in this group primarily exhibit negative emotions, have poor eye contact and do not utilise hand or body gestures during the session.

These rules provide a guideline for evaluating student performance based on their non-verbal behaviours and emotions, thereby allowing for the assignment of scores within the defined groups.

#### 4.1.11. Active Learning

In the traditional machine learning setup, the data are partitioned into training and testing data. The training data are used for training the models by exploiting the labelled data, whilst the testing data are used for testing the models. However, for a robotic scoring approach similar to our RPSS, the testing data are captured by real-time sensors, and the quality of the record can be controlled by the robot. Therefore, changing the position of the robot can improve the prediction performance. Active learning refers to the process of exploiting the robot to improve the quality of the record. In the context of robotic presentation scoring, active learning can be mathematically defined as follows.

Assuming that we have a controller C that produces a sample $x_{t}=C(u_{t})$, where $u_{t}$ denotes the control action, and assuming that we have a classification model $y_{t}^{\prime }=f(x_{t}.w)$, where $y_{t}^{\prime }$ denotes the prediction by the classifier, we also assume that $\theta (x_{t})$ is a confidence measure from the classification of the sample $x_{t}$. Active learning is the optimisation process that changes the value of $x_{t}$ until the value of $\theta (x_{t})$ reaches its maximum.

(8) $$y_{t}^{\prime }=f(C\left(argmax\left(\theta \left(C\left(u_{t}\right)\right)\right)\right),w)$$

Assuming that $u\in [u_{min}\;u_{max}]$, the optimisation is solved by performing a linear search in the interval of $\left[u_{min}\;u_{max}\right]$ to maximise the value of θ. Upon obtaining the value of $u_{t}$ that maximises the value of θ, we use its associated sample of $x_{t}=C(u_{t})$ for predicting the class $y_{t}^{\prime }$. The pseudocode of active learning is presented in Algorithm 5.
**Algorithm 5:** Pseudocode of active learning for RPSS**Input**:    (1) w weights   (2) f classification   (3) $u\in [u_{min}\;u_{max}]$ control interval **Output**:    $y_{t}^{\prime }$
**Start algorithm**: *1:Initiate an empty list*
Θ ***2: For***
*each*
u
*between*
$\left[u_{min}\;u_{max}\right]$*** do*** *3:  Calculate the corresponding value of*
$x_{t}=C(u_{t})$ *4:  Add the value of*
$\theta (x_{t})$
*to the list of*
Θ
*with the corresponding*
$x_{t}$ *5: **End for*** *6: Find the value of*
$x_{t}$
*that is associated with the maximum value of*
θ
*inside*
Θ
*using linear search.* *7: Return*
$y_{t}^{\prime }=f(x_{t}.w)$ 8: **End algorithm.**

## 5. Experimental Works and Analysis

This section presents the datasets description, the decision-making results of our RPSS, and the integration of the scoring approach with the selected four multi-model components.

### 5.1. Datasets

The study draws upon a diverse range of datasets to facilitate the comprehensive evaluation of presentation skills. To offer a detailed introduction, we can categorise these datasets as follows: 1—Custom Presentation Dataset: A substantial portion of our dataset was custom-built to address the intricate aspects of eye contact, facial emotions, and hand and body movement. This dataset is unique to our research and specifically tailored for the study of presentation skills and non-verbal cues. 2—DAiSEE Dataset [16]: To investigate facial emotions, we incorporated the DAiSEE dataset. This established dataset comprises a wide array of facial expressions, providing a robust foundation for the analysis of emotional responses during presentations. 3—TEDx Talks videos [17]: For the evaluation of body movement, the TEDx Talks videos, alongside our custom dataset, were employed as the gold standard for optimal presentation behaviour. This dataset encompasses real-world TEDx Talks, which serve as a valuable reference for assessing body movement patterns during public speaking engagements.

By amalgamating these datasets, the study ensures a comprehensive and multifaceted examination of presentation skills, encompassing aspects of eye contact, hand movement, facial emotions, and body movement. This approach enhances the depth and validity of the research findings, providing a well-rounded perspective on non-verbal communication within the context of presentations. A table of the statistical description of each of the datasets is presented in Table 11.

### 5.2. Decision Making Results

Rules-based AHP is applied to assign scores to our four scenarios. We integrate this scoring approach into RPSS, whose architecture includes the TurtleBot V3 robot (Wuhan Jingtian Electric Appliance Co., Ltd., Wuhan, China) equipped with the NVIDIA Jetson Nano™ Developer Kit (NVIDIA, Taipei, Taiwan), Intel RealSense Depth Camera (D435) and a 7-inch screen brand (Brand Chuanglebo, Changsha, China) to display the score. The robot localises its optimal position using the linear search technique based on the confidence of the eye contact and facial emotions of each presenter. The results of our four multi-classification models are discussed in the following sub-sections.

#### 5.2.1. Eye Contact

For eye contact, we compared our used DeepEC approach and the benchmark, which is heuristic [47]. Table 12 represents the classification metrics for the DeepEC approach overall. The accuracy for both classes, “No-Eye contact” and “Eye contact”, is 0.73%. The precision for “No-Eye contact” is 0.76%, indicating a high percentage of correctly classified instances. The recall for “No-Eye contact” is 0.70%, indicating the ability of the model to correctly identify instances of “No-Eye contact” out of the total instances available. The f1 score for “No-Eye contact” is 0.73%, which is a balanced measure of precision and recall. The support for “No-Eye contact” is 10,764, representing the number of instances in that class. Similarly, for the “Eye contact” class in Table 1, the precision is 0.70%, indicating a relatively high percentage of correctly classified instances. The recall is 0.76%, indicating the model’s ability to correctly identify instances of “Eye contact” out of the total instances available. The f1 score for “Eye contact” is 0.73%, which represents a balanced measure of precision and recall. The support for “Eye contact” is 9938, indicating the number of instances in that class.

Table 13 represents the classification metrics for the heuristic method overall. The accuracy for both classes, “No-Eye contact” and “Eye contact”, is 0.52%, indicating a relatively low overall accuracy. The precision for “No-Eye contact” is 0.58%, and the recall is 0.41%. The f1 score for “No-Eye contact” is 0.48%. These metrics suggest that the heuristic method struggles to accurately classify instances of “No-Eye contact”. The support for “No-Eye contact” is 10,198. For the “Eye contact” class in Table 13, the precision is 0.48%, and the recall is 0.65%. The f1 score for “Eye contact” is 0.55%. These metrics indicate that the heuristic method performs relatively better in identifying instances of “Eye contact” compared to “No-Eye contact”. The support for “Eye contact” is 8729.

Overall, the classification metrics demonstrate that DeepEC achieves higher accuracy, precision, recall, and f1 scores for both classes compared to the heuristic method. DeepEC shows better performance in correctly identifying instances of “No-Eye contact” and “Eye contact” based on the provided metrics, highlighting its superiority in accurately detecting eye contact. Hence, DeepEC was selected as a sub-component in the scoring system for eye contact detection.

#### 5.2.2. Face Emotions

The analysis of five deep learning models for five categories of facial expressions (boredom, engagement, confusion, frustration and delight) demonstrates the superior performance of EfficientNet compared with other models, such as Xception, Inception, ResNet and MobileNet. These models are initially trained on the Daisee dataset and finetuned on a custom dataset to introduce the delight category.

Table 14 presents the comparison results. The F1 scores of EfficientNet across all five classes of the custom dataset demonstrate its superior performance compared with other models. Specifically, EfficientNet achieves high F1 scores for boredom (0.62), engagement (0.81), confusion (0.69) and frustration (0.62), but its F1 score for delight is relatively low (0.44), thereby suggesting some difficulty in recognising this particular expression.

EfficientNet obtains an overall efficiency of 0.69, which indicates its ability to accurately classify facial expressions in the majority of the cases. The macro average F1 score is 0.64, whilst the weighted average F1 score is 0.68, which further reflects the efficient performance of the model across all classes, with higher weights given to the more populated classes.

As presented in Table 15, the confusion matrix for EfficientNet provides further insights into its classification performance. The matrix shows the distribution of predicted labels versus actual labels for each class. EfficientNet exhibits high precision in identifying boredom and engagement, as indicated by the relatively high values along the diagonal for these classes. However, there seems to be some confusion between boredom and frustration and between confusion and frustration. The model struggles the most in accurately classifying delight, as indicated by the lower values along the diagonal for this class.

In sum, EfficientNet demonstrates superior performance in facial expression classification compared with the other models, as reflected in its high accuracy and F1 scores across most classes. However, this model demonstrates some limitations in accurately recognising the delight expression and encounters confusion between certain classes. Further analysis and potential improvements may be necessary to address these problems.

#### 5.2.3. Hand Movement

In the “Without Kalman-Filter” method, the model achieves a precision of 0.53 and recall of 0.94 for non-movement instances in score A, indicating its accurate identification of non-movement instances and potential misclassification of some movement instances. The precision (0.94) and recall (0.53) for movement instances are similar. For score D, the model correctly classifies all non-movement instances. The performance metrics in Table 16 visualise the performance of the model in scores A and D. With the inclusion of the Kalman filter in the “With Kalman-Filter” method, the precision and recall of the model for non-movement instances in score A are improved. Specifically, the model precision remains at 0.53, whilst its recall increases to 0.95, thereby indicating an improved identification of non-movement instances. Similarly, the precision for movement instances increases to 0.95, whilst the recall slightly decreases to 0.52. The model maintains its perfect accuracy in classifying non-movement instances in score D. The performance metrics in Table 17 visualise the performance of the model using the Kalman-Filter method in scores A and D. The “Hybrid” approach, which combines the Kalman filter with conditional activation, achieves the same precision and recall values for non-movement instances in score A. However, some improvements are observed in score D. Specifically, the model precision remains at 1, indicating no false positives for non-movement instances, whilst its recall increases to 0.72. The performance metrics in Table 18 visualise the performance of this model in scores A and D.

Overall, the inclusion of the Kalman filter improves the accuracy of detecting hand movement. The hybrid method further enhances the performance of the model in accurately identifying non-movement instances in score D.

#### 5.2.4. Body Movement

In the “Without Kalman-Filter” method, the model achieves a precision of 0.62 and recall of 0.32 for no-body movement instances in Video1_TEDx, thereby indicating that this model accurately identifies some instances of no-body movement but may misclassify a significant number of these instances. For body movement instances, the model achieves a precision of 0.4, indicating a relatively low accuracy, and a recall of 0.7, indicating an improved ability to detect body movement. Similar performance patterns are observed in Video2_TEDx. The performance metrics in Table 19 visualise the performance of this model in detecting body movement without Kalman filtering in TEDx video streams.

The inclusion of the Kalman filter in the “With Kalman-Filter” method results in slight improvements in the precision and recall values of the model for both no-body and body movement instances in Video1_TEDx and Video2_TEDx. However, its overall performance remains similar to that of the model without Kalman filtering. The performance metrics in Table 20 visualises the performance of this model in detecting body movement.

The “Hybrid” approach achieves the same precision and recall values for both no-body movement and body movement instances in Video1_TEDx and Video2_TEDx, thereby indicating the lack of any significant improvements in the performance of the model. The performance metrics in Table 21 visualises the performance of this model in detecting body movement.

### 5.3. Integrated the Scoring Approach and the Selected Components

The best models are then selected for each criterion as follows:

1. Body Movement: Amongst the three methods, the Hybrid method shows the most promise, given its relatively high precision, recall, F1 score and accuracy for both Videos 1 and 2 from the TEDx dataset. By combining the benefits of the “With Kalman-Filter” and “Without Kalman-Filter” methods, this method achieves an improved performance in detecting body movement.
2. Hand Movement: The model incorporating Kalman filtering achieves the highest precision, recall, F1 score and accuracy for both scores A and D in the hand movement classification task.
3. Eye Contact: DeepEC is selected, which is a supervised model.
4. Face Emotion: EfficientNet outperforms the other models (Xception, Inception, ResNet and MobileNet) in classifying facial expressions as reflected in its higher F1 scores and accuracy across the five emotion categories.

**Figure 6:**
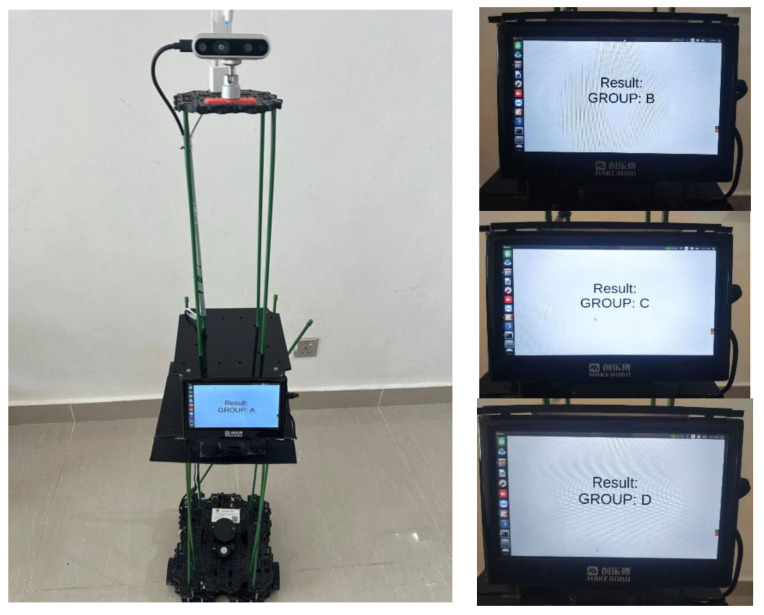
The scoring results in the real-time case study in the lab environment.

We then evaluate our proposed RPSS by conducting a real-world case study from two perspectives, namely user experience evaluation and experts’ evaluation scoring, as will be discussed in the following section. Figure 6 illustrates some of the student’s scoring results of the real-time case study. The results for groups A–D have been discussed in detail in the section on the rule-based scoring model. The results are displayed on the screen, and the screen Chinese term denotes the brand name of the screen, ‘Chuang lebo’.

#### 5.3.1. Case Study: User Experience Evaluation

We adopt an interactive evaluation process to gather insightful user opinions on RPSS. The users actively engage with RPSS by delivering their presentations in front of the robot, thereby experiencing its functionalities in real time. Fourteen users delivered their presentations in front of the robot in two states, namely traditional learning (with a stationary camera) and active learning (the robot localises its optimal position to that of the presenter).

The users provide overwhelmingly positive feedback on RPSS, with the majority of the functionalities being rated as “extremely useful” or “very useful”. Around 75% of these users have attributed the effectiveness of RPSS in capturing and evaluating body movement to its scoring approach and highlighted the robustness of its multi-model analysis approach. Meanwhile, 58.3% and 66.7% of these users have claimed that the hand movement and facial expression evaluations of RPSS are extremely accurate, respectively, and 50% and 41.7% of these users have rated the fairness of the AHP-based weighting scoring approach as extremely fair and very fair, respectively. They also praise the feedback mechanism and user interface of RPSS, which they describe as accurate and user-friendly. Overall, RPSS is a comprehensive, reliable and impactful tool for automated presentation evaluation that shows high potential for widespread adoption. The percentage distribution of user ratings is presented in Table 22.

#### 5.3.2. Robot Scoring Group vs. Experts Scoring Group

In our study, the scores generated by the robot were evaluated by comparing them with expert evaluations, which were annotated scores ranging from A to D for the full presentation video based on video-level annotations. Five experts were engaged to evaluate and score the presentations of the participants [17].

The data presents an intriguing comparison between the scoring decisions made by a robotic approach and those made by human tutors. The scoring is categorised into different groups (A, B, C or D) for each presenter under two different scoring methods: Traditional and Active learning, as illustrated in Table 23.

In the Traditional scoring method, the robotic approach and the human tutors show a high level of agreement for most presenters. For instance, Presenters 2, 3, 4, 5, 8, 10, 11, 12 and 14 all received unanimous group classifications from both the robot and the tutors. However, discrepancies do exist. For Presenter 1, the robot placed the individual in group C, while the tutors’ average also indicated group C, but one tutor deviated by placing the presenter in group B and another in group A. Similarly, for Presenter 6 and Presenter 9, the robot’s decision was mostly in line with the tutors, but there were minor deviations. Presenter 13 showed the most significant discrepancy, with the robot placing the presenter in group B while the tutors were divided between groups A and B. The total average agreement in the traditional method stands at 90%.

In the active learning scoring method, the agreement between the robot and the tutors is even higher. For Presenters 2, 3, 5, 6, 8, 9, 10, 11, 12 and 14, both the robot and the tutors were in complete agreement. The only notable discrepancies were for Presenter 4, where the robot placed the presenter in group B, while one tutor deviated by placing them in group A. Presenter 7 also showed a similar pattern, with the robot and most tutors placing the presenter in group A. The total average agreement in the Active learning method is remarkably high at 99%.

Overall, the robotic approach shows a high level of agreement with human tutors in both scoring methods, with the Active5 method showing an almost perfect alignment. These findings suggest that the robotic approach is highly reliable and could serve as an effective tool for automated presentation scoring. The minor discrepancies that do exist could be attributed to the subjective nature of human judgment, which may capture nuances that the robotic approach is not programmed to consider.

The high level of agreement between the robotic approach and human tutors, particularly in the Active5 method, lends credibility to the potential integration of such automated approaches in educational settings. Therefore, the robotic approach could be considered a reliable tool for presentation scoring, complementing human expertise.

## 6. Conclusions and Future Works

The proposed RPSS aims to assess and enhance the presentation skills of students using multiple criteria. This approach incorporates advanced technologies and models to evaluate the hand movement, body movement, eye contact and facial expressions of presenters. RPSS employs EfficientNet for facial expression analysis, DeepEC for eye contact detection and Kalman filters for smooth tracking and prediction of movement.

The models for RPSS are trained on appropriate datasets, including the Daisee dataset for facial expressions and a custom dataset to introduce the delight category. Fine-tuning and augmentation techniques are also applied to improve their performance.

During the evaluation phase, several students are invited to deliver their presentations in front of a robot, and their scores generated by RPSS are compared with those generated via human evaluations. User feedback is then collected via a questionnaire survey and in-depth interviews. These users have an overwhelmingly positive perception of RPSS, particularly in terms of its perceived usefulness and feedback. They also acknowledge the potential of RPSS for improving their presentation skills and gaining valuable insights.

Although some users have raised their concerns, such as their awareness of being recorded and the limitations in their environment, they describe RPSS as a useful approach that provides valuable learning experiences. These findings highlight the effectiveness of RPSS and the significance of incorporating advanced technologies and models for comprehensive presentation skill assessment and improvement.

In terms of classification performance, the active learning-based RPSS demonstrates its superiority over traditional learning-based RPSS. Furthermore, an intriguing comparison between the scoring decisions made by a robotic approach and those made by human tutors has shown that the total average agreement in the traditional method stands at 90%, and the total average agreement in the active learning method is remarkably high at 99%.

As a limitation, our current hand gesture detection algorithm primarily focuses on the shoulder, elbow, and wrist joints, which may result in false negatives for gestures that are executed predominantly with the hand and wrist. This limitation could lead to an underestimation of the nuanced hand gestures commonly used in presentations, potentially affecting the overall accuracy of our gesture detection framework.

Future work on RPSS should focus on integrating audio analysis, incorporating real-time feedback, developing personalised learning paths, expanding the dataset and enhancing the user interface, which would further strengthen the assessment capabilities of this approach and provide a comprehensive and effective platform for developing exceptional presentation skills. Future research could involve investigating the performance and scalability of the proposed methods in multi-robot settings or larger presentation venues, building upon the primary focus on a single robot for presentation scoring.

## Figures and Tables

**Table 1:** Summary of existing approaches used for presentation scoring.

Article	Sensing		Face Emotion Classifier	Eye Contact Classifier	Hand Gesture Classifier	Body Movement	Robot	Overall Scoring
	Depth	Video						
[17]	×	√	×	√	√	√	×	Regression methods to predict the score (awarded by tutors)
[11]	×	√	√	×	×	√	×	×
[18]	×	√	√	√	×	×	×	×
[13]	×	√	×	√	√	√	×	Weighted sum according to the weight of each factor
Ours	√	√	√	√	√	√	√	AHP Rule-based

Note: The symbol “×” indicates that a particular factor has not been employed in the research, whilst “√” indicates that this factor has been employed.

**Table 2:** Summary of the existing approaches used for basic and learning-centred emotion classification models.

Refs.	Face	Eye Contact	Body and Hand	Basic Emotion	Learning Emotions	Feature Extraction	Classifier	Ensemble Learning	Deep Learning Model
[19]	√	×	×	0	2	Gabor wavelets	SVM	×	×
[20]	√	×	×	0	2	Histogram of oriented gradient (HOG); Local binary patterns (LBP)	SVM/FC	×	VGG16
[21]	√	√	×	7	1	Viola–Jones algorithm as face detector	Softmax	×	Eye contact: CNN; face: mini-Xception
[22]	√	×	×		1	OpenFace detects and tracks	Softmax	XGBoost classifier for 2 models.	
[23]	√	×	√	8	0	OpenFace 2 toolkit; for body: OpenPose	Softmax	×	For face: Resnet-50, AffectNet; for body: DNN with global temporal average pooling (GTAP)
[24]	√	√	×	6	0	OpenCV and Dlib library	Softmax/SVM	×	CNN
[25]	√	×	×	6	0	Feature sparseness of the FC input; proposed L2 sparseness	Softmax	×	VGG, ResNet
[26]	√	×	×	6	0	Haar features detects.	Softmax	×	AlexNet, GoogleNet, ResNet structures
[27]	√	×	×	7	0	Integrated OpenCV, Dlib, Mtcnn, and Tinyface. VGG16 model	Softmax	×	Multi-layer perceptron (MLP) classifier with Adam optimiser
[28]	√	×	×	7	0	Tiny_dnn	×	nViso and Oxford approaches	×
[29]	√	×	×	7	0	HOG face detector	Softmax	×	CNN with unsupervised training gradual greedy layer-wise algorithm (Gradual-GLW)
[30]	×	√	×	0	0	Unsupervised eye contact pipeline/CNN Model	Binary support vector machine (SVC) classifier/Softmax	×	CNN Model
[31]	×	√	×	0	0	ResNet50	Softmax	×	CNN Model
Ours	√	√	√	0	4	Face features using MediaPipe	Softmax	×	EfficientNet; DeepEC

Note: The symbol “×” indicates that a particular factor has not been employed in the research, whilst “√” indicates that this factor has been employed.

**Table 3:** Overview of factors used in previous studies to evaluate students’ presentation skills or human–machine interactions.

Refs.	Application	Facial Expression	Eye Contact	Hand Gesture	Body Posture	ID	Gender	Duration	Slides	Audio
[24]	Learner’s emotion recognition model in online learning.	√	×	×	×	×	×	×	×	×
[26]	Facial expression recognition in robotics.	√	×	×	×	×	×	×	×	×
[28]	Facial expression recognition for robots.	√	×	×	×	×	×	×	×	×
[29]	Facial expression recognition for robots.	√	×	×	×	×	×	×	×	×
[16]	Learner’s emotion recognition model in online learning.	√	×	×	×	×	×	×	×	×
[19]	Learner’s emotion recognition model in online learning.	√	×	×	×	×	×	×	×	×
[20]	Learner’s emotion recognition model in online learning.	√	×	×	×	×	×	×	×	×
[21]	Learner’s emotion recognition model in online learning.	√	√	×	×	×	×	×	×	×
[22]	Learner’s emotion recognition models for TA robot.	√	√	×	×	×	×	×	×	×
[11]	Learner’s emotion recognition models in presentation sessions.	√	×	√	√	√	√	√	×	×
[23]	Learner’s emotion recognition models for robots.	√	×	√	√	×	×	×	×	×
[15]	Learner’s emotion recognition model in online and classroom learning.	√	×	√	√	×	×	×	×	×
[17]	Learner’s emotion recognition system in presentation sessions.	×	√	√	√	×	×	×	√	√
[13]	Learner’s emotion recognition models in presentation sessions.	×	√	√	√	×	×	×	√	√
[30]	Eye contact detection in human–computer interaction.	×	√	×	×	×	×	×	×	×
[31]	Eye contact detection in human–computer interaction.	×	√	×	×	×	×	×	×	×
[35]	Real-time human action detection.	×	×	√	√	×	×	×	×	×
[36]	Human–robot interaction for the human body avoidance scenario.	×	×	√	√	×	×	×	×	×

Note: The symbol “×” indicates that a particular factor has not been employed in the research, whilst “√” indicates that this factor has been employed.

**Table 4:** Sample questions asked to experts in the Fuzzy Delphi survey for presentation skills scoring.

Factor	Question
(1) Facial expressions	From your point of view, facial expressions or emotions are classified as
(2) Eye contact	Eye contact is classified as
(3) Hand gestures and movements	Hand gestures and movements are classified as

**Table 5:** Definition of factors based on the literature.

Refs.	Factor	Definition
[16]	Facial expression	Indicates facial expressions that are associated with certain emotions, such as happiness and seriousness.
[31]	Eye contact	A form of non-verbal communication that can have a large influence on social behaviours.
[15]	Hand gesture and movement	A form of non-verbal communication where visible bodily actions send messages.
[36]	Body posture and movement	A form of non-verbal communication where the whole body is used to send out a message, which can be a critical indicator of attitude.
[11]	ID	Information used by computer systems to represent a person
[11]	Gender	The distinction between gender identities, whether male or female.
[11]	Duration	The time or period elapsed during a presentation.
[17]	Slides	The content of slides used in a presentation.
[17]	Audio	A representation of spoken sound data.

**Table 6:** Gaussian fuzzy sets for importance levels.

Scale	Level of Importance	Mean (µ)	Standard Deviation (σ)
1	Extremely strongly non-important	0.0	0.09
2	Strongly non-important	0.1	0.09
3	Non-important	0.3	0.09
4	Moderately important	0.5	0.09
5	Important	0.7	0.09
6	Strongly important	0.9	0.09
7	Extremely strongly important	1.0	0.09

**Table 7:** Linguistic terms used for criteria weighting in the Fuzzy Delphi survey.

Factor	Weight of Fuzzy Delphi	Decision
Facial expression	0.9010	Select
Eye contact	0.9048	Select
Hand gesture and movement	0.7999	Select
Body posture and movement	0.7750	Select
ID	0.7750	Select
Gender	0.57	Select
Duration	0.8392	Select
Slides	0.8931	Select
Audio	0.8628	Select

**Table 8:** Weights of the four criteria based on AHP.

Eye Contact	Facial Expressions	Hand Movement	Body Movement
0.479307	0.283206	0.146526	0.090961

**Table 9:** Nationalities of the participants.

Nationality	Number of Participants
Malay	6
Chinese	4
Indian	2
Bangladeshi	5
Iraqi	5

**Table 10:** Ages of the participants.

Number of Participants	Age Range
11	20–24
6	25–29
5	30–40

**Table 11:** A statistical overview of the used datasets.

Name	Number of Videos	Number of Presenters	Period of Video
Custom Presentation Dataset	88	22	5 h and 30 min
DAiSEE Dataset	9068	112	25 h
TEDx-based videos	8	7	39 min

**Table 12:** Overall classification results using the Deep EC model.

Class/State	Accuracy	Precision	Recall	F1 Score	Support
No-Eye contact	0.729688	0.758452	0.704478	0.730469	10,764
Eye contact	0.729688	0.702821	0.756993	0.728902	9938

**Table 13:** Overall classification results using the heuristic method.

Class/State	Accuracy	Precision	Recall	F1 Score	Support
No-Eye contact	0.519787	0.575928	0.412434	0.480658	10,198
Eye contact	0.519787	0.484515	0.645206	0.553432	8729

**Table 14:** F1 scores of each model implied on the five classes of the custom dataset.

Face Expression	Xception	Inception	ResNet	MobileNet	EfficientNet
Boredom	0.62	0.58	0.01	0.36	**0.62**
Engagement	0.84	0.78	0.54	0.55	**0.81**
Confusion	0.60	0.64	0.33	0.08	**0.69**
Frustration	0.52	0.51	0.34	0.27	**0.62**
Delight	0.38	0.08	0.00	0.00	**0.44**
Accuracy	0.64	0.61	0.40	0.40	**0.69**
Macro avg	0.59	0.52	0.25	0.25	**0.64**
Weighted avg	0.60	0.69	0.33	0.33	**0.68**

**Table 15:** EfficientNet confusion matrix.

	Boredom	Engaged	Confusion	Frustration	Delight
Boredom	0.689	0.051	0.073	0.144	0.043
Engaged	0.06	0.723	0.038	0.048	0.131
Confusion	0.097	0.016	**0.756**	0.124	0.008
Frustration	0.244	0.036	0.089	0.612	0.019
Delight	0.141	0.085	0.092	0.106	0.577

**Table 16:** Performance metrics of hand movement without Kalman filter for scores (A) and (D).

Hand	Class	Precision	Recall	F1 Score	Accuracy
Score A	Non-movement	0.53	0.94	0.68	0.68
	Movement	0.94	0.53	0.68	0.68
Score D	Non-movement	1	1	1	1
	Movement	0	0	0	1

**Table 17:** Performance metrics of hand movement with Kalman filter for scores (A) and (D).

Hand	Class	Precision	Recall	F1 Score	Accuracy
Score A	Non-movement	0.53	0.95	0.68	0.68
	Movement	0.95	0.52	0.67	0.68
Score D	Non-movement	1	1	1	1
	Movement	0	0	0	1

**Table 18:** Performance metrics of hand movement with hybrid method for scores (A) and (D).

Hand	Class	Precision	Recall	F1 Score	Accuracy
Score A	Non-movement	0.53	0.94	0.68	0.68
	Movement	0.94	0.53	0.68	0.68
Score D	Non-movement	1	0.72	0.84	0.72
	Movement	0	0	0	0.72

**Table 19:** Performance metrics of body movement without Kalman filter for TEDx video streams.

Body	Class	Precision	Recall	F1 Score	Accuracy
Video1_TEDx	No-body movement	0.62	0.32	0.43	0.47
	Body movement	0.4	0.7	0.51	0.47
Video2_TEDx	No-body movement	0.57	0.38	0.46	0.56
	Body movement	0.55	0.73	0.63	0.56

**Table 20:** Performance metrics of body movement with Kalman filter for TEDx video streams.

Body	Class	Precision	Recall	F1 Score	Accuracy
Video1_TEDx	No-body movement	0.64	0.36	0.46	0.49
	Body movement	0.41	0.68	0.51	0.49
Video2_TEDx	No-body movement	0.56	0.39	0.46	0.55
	Body movement	0.54	0.71	0.62	0.55

**Table 21:** Performance metrics of body movement with the hybrid method for TEDx video streams.

Body	Class	Precision	Recall	F1 Score	Accuracy
Video1_TEDx	No-body movement	0.62	0.32	0.43	0.47
	Body movement	0.4	0.7	0.51	0.47
Video2_TEDx	No-body movement	0.57	0.38	0.46	0.56
	Body movement	0.55	0.73	0.63	0.56

**Table 22:** Percentage distribution of user ratings across various criteria.

Questions	The Five Scales				
	1	2	3	4	5
1	0%	0%	8.3%	16.7%	75%
2	0%	0%	8.3%	33.3%	58.3%
3	0%	0%	0%	25%	75%
4	0%	0%	0%	25%	75%
5	0%	0%	0%	33.3%	66.7%
6	0%	0%	0%	50%	50%
7	0%	0%	8.3%	25%	66.7%
8	0%	0%	8.3%	41.7%	50%
9	0%	0%	0%	33.3%	66.7%
10	0%	0%	0%	41.7%	58.3%
11	0%	0%	0%	33.3%	66.7%
12	0%	0%	8.3%	25%	66.7%

**Table 23:** Robot scoring versus expert scoring in traditional and active learning approaches.

	Traditional							Active5						
	Robot	Tutor1	Tutor2	Tutor3	Tutor4	Tutor5	Avg	Robot	Tutor1	Tutor2	Tutor3	Tutor4	Tutor5	Avg
Presenter 1	group:C	group:B	group:A	group:C	group:C	group:C	60%	group:A	group:A	group:A	group:A	group:A	group:A	100%
Presenter 2	group:B	group:B	group:B	group:B	group:B	group:B	100%	group:A	group:A	group:A	group:A	group:A	group:A	100%
Presenter 3	group:D	group:D	group:D	group:D	group:D	group:D	100%	group:B	group:B	group:B	group:B	group:B	group:B	100%
Presenter 4	group:D	group:D	group:D	group:D	group:D	group:D	100%	group:B	group:A	group:B	group:B	group:B	group:B	80%
Presenter 5	group:A	group:A	group:A	group:A	group:A	group:A	100%	group:A	group:A	group:A	group:A	group:A	group:A	100%
Presenter 6	group:B	group:B	group:B	group:C	group:B	group:B	80%	group:B	group:B	group:B	group:B	group:B	group:B	100%
Presenter 7	group:B	group:A	group:B	group:B	group:B	group:B	80%	group:A	group:A	group:A	group:A	group:A	group:A	100%
Presenter 8	group:C	group:C	group:C	group:C	group:C	group:C	100%	group:B	group:B	group:B	group:B	group:B	group:B	100%
Presenter 9	group:B	group:B	group:B	group:B	group:A	group:B	80%	group:B	group:B	group:B	group:B	group:B	group:B	100%
Presenter 10	group:B	group:B	group:B	group:B	group:B	group:B	100%	group:B	group:B	group:B	group:B	group:B	group:B	100%
Presenter 11	group:D	group:D	group:D	group:D	group:D	group:D	100%	group:B	group:B	group:B	group:B	group:B	group:B	100%
Presenter 12	group:C	group:C	group:C	group:C	group:C	group:C	100%	group:B	group:B	group:B	group:B	group:B	group:B	100%
Presenter 13	group:B	group:A	group:B	group:B	group:B	group:A	60%	group:B	group:B	group:B	group:B	group:B	group:B	100%
Presenter 14	group:D	group:D	group:D	group:D	group:D	group:D	100%	group:C	group:C	group:C	group:C	group:C	group:C	100%
						Total Avg	90%						Total Avg	99%

## Data Availability

Data is not available for privacy restriction.

## References

[1] Ramli I.S.M., Maat S.M., Khalid F. (2022). The Design of Game-Based Learning and Learning Analytics. Cypriot J. Educ. Sci..

[2] Saini M.K., Goel N. (2019). How smart are smart classrooms? A review of smart classroom technologies. ACM Comput. Surv..

[3] Hussin M., Said M.S., Mohd Norowi N., Husin N.A., Mustaffa M.R. (2021). Authentic Assessment for Affective Domain through Student Participant in Community Services. Asia-Pac. J. Inf. Technol. Multimed..

[4] Sun Z., Li Z., Nishimorii T. Development and assessment of robot teaching assistant in facilitating learning. Proceedings of the 6th International Conference of Educational Innovation through Technology EITT.

[5] Alshammari R.F.N., Arshad H., Rahman A.H.A., Albahri O.S. (2022). Robotics Utilization in Automatic Vision-Based Assessment Systems from Artificial Intelligence Perspective: A Systematic Review. IEEE Access.

[6] Ahmed H., La H.M. Education-Robotics Symbiosis: An Evaluation of Challenges and Proposed Recommendations. Proceedings of the 2019 9th IEEE Integrated STEM Education Conference (ISEC).

[7] Ahmed Soliman S. (2019). Efficiency of an Educational Robotic Computer-mediated Training Program for Developing Students’ Creative Thinking Skills: An Experimental Study. Arab. World Engl. J..

[8] Abd Rahman A.H., Sulaiman R., Sani N.S., Adam A., Amini R. (2019). Evaluation of peer robot communications using cryptoros. Int. J. Adv. Comput. Sci. Appl..

[9] Hsieh Y.Z., Lin S.S., Luo Y.C., Jeng Y.L., Tan S.W., Chen C.R., Chiang P.Y. (2020). ARCS-assisted teaching robots based on anticipatory computing and emotional Big Data for improving sustainable learning efficiency and motivation. Sustainability.

[10] Yoshino K., Zhang S. Construction of Teaching Assistant Robot in Programming Class. Proceedings of the 2018 7th International Congress on Advanced Applied Informatics, IIAI-AAI.

[11] Fekry A., Dafoulas G., Ismail M. Automatic detection for students behaviors in a group presentation. Proceedings of the ICCES 2019: 2019 14th International Conference on Computer Engineering and Systems.

[12] Bhole G.P., Deshmukh T. (2018). Multi-criteria decision making (MCDM) methods and its applications. Int. J. Res. Appl. Sci. Eng. Technol..

[13] Ochoa X., Domínguez F., Guamán B., Maya R., Falcones G., Castells J. (2018). The RAP system: Automatic feedback of oral presentation skills using multimodal analysis and low-Cost sensors. ACM Int. Conf. Proc. Ser..

[14] Shahrim K., Abd Rahman A.H., Goudarzi S. (2022). Hazardous Human Activity Recognition in Hospital Environment Using Deep Learning. IAENG Int. J. Appl. Math..

[15] Ashwin T.S., Guddeti R.M.R. (2020). Affective database for e-learning and classroom environments using Indian students’ faces, hand gestures and body postures. Future Gener. Comput. Syst..

[16] Gupta A., D’Cunha A., Awasthi K., Balasubramanian V. (2016). DAiSEE: Towards User Engagement Recognition in the Wild. arXiv.

[17] Haider F., Koutsombogera M., Conlan O., Vogel C., Campbell N., Luz S. (2020). An Active Data Representation of Videos for Automatic Scoring of Oral Presentation Delivery Skills and Feedback Generation. Front. Comput. Sci..

[18] Tun S.S.Y., Okada S., Huang H.H., Leong C.W. (2023). Multimodal Transfer Learning for Oral Presentation Assessment. IEEE Access.

[19] Daud S.A.A., Lutfi S.L. Towards the detection of learner’s uncertainty through face. Proceedings of the 2016 4th International Conference on User Science and Engineering, i-USEr.

[20] Shi Z., Zhang Y., Bian C., Lu W. Automatic academic confusion recognition in online learning based on facial expressions. Proceedings of the 14th International Conference on Computer Science and Education, ICCSE.

[21] Sharma P., Joshi S., Gautam S., Maharjan S., Filipe V. (2019). Student Engagement Detection Using Emotion Analysis, Eye Tracking and Head Movement with Machine Learning Cabral Reis Universidade de Tras-os-Montes e Alto Douro, Vila Real, Portugal Institute of Electronics and Informatics Engineering of Aveiro, Port. arXiv.

[22] Liao D., Wu T., Chen Y. An interactive robot for fatigue detection in the learning process of children. Proceedings of the 2017 2nd International Conference on Advanced Robotics and Mechatronics (ICARM).

[23] Filntisis P.P., Efthymiou N., Koutras P., Potamianos G., Maragos P. (2019). Fusing body posture with facial expressions for joint recognition of affect in child—Robot interaction. IEEE Robot. Autom. Lett..

[24] Li G., Wang Y. Research on leamer’s emotion recognition for intelligent education system. Proceedings of the 2018 IEEE 3rd Advanced Information Technology, Electronic and Automation Control Conference, IAEAC.

[25] Xie W., Jia X., Shen L., Yang M. (2019). Sparse deep feature learning for facial expression recognition. Pattern Recognit..

[26] He Z., Jin T., Basu A., Soraghan J., Di Caterina G., Petropoulakis L. Human emotion recognition in video using subtraction pre-processing. Proceedings of the ICMLC’19: Proceedings of the 2019 11th International Conference on Machine Learning and Computing.

[27] Khanh T.L.B., Kim S.H., Lee G., Yang H.J., Baek E.T. (2021). Korean video dataset for emotion recognition in the wild. Multimed. Tools Appl..

[28] Espinosa-Aranda J.L., Vallez N., Rico-Saavedra J.M., Parra-Patino J., Bueno G., Sorci M., Moloney D., Pena D., Deniz O. (2018). Smart doll: Emotion recognition using embedded deep learning. Symmetry.

[29] Webb N., Ruiz-Garcia A., Elshaw M., Palade V. Emotion Recognition from Face Images in an Unconstrained Environment for usage on Social Robots. Proceedings of the International Joint Conference on Neural Networks.

[30] Müller P., Huang M.X., Zhang X., Bulling A. Robust eye contact detection in natural multi-person interactions using gaze and speaking behaviour. Proceedings of the Eye Tracking Research and Applications Symposium (ETRA).

[31] Chong E., Clark-whitney E., Southerland A., Stubbs E., Miller C., Ajodan E.L., Silverman M.R., Lord C., Rozga A., Jones R.M. (2020). Is As Accurate as Human Experts. Nat. Commun..

[32] Sahebi I.G., Masoomi B., Ghorbani S. (2020). Expert oriented approach for analyzing the blockchain adoption barriers in humanitarian supply chain. Technol. Soc..

[33] Nayak S., Pattanayak S., Choudhury B.B., Kumar N., Behera H.S., Nayak J., Naik B., Pelusi D. (2020). Selection of Industrial Robot Using Fuzzy Logic Approach. Proceedings of the 5th International Conference on Computational Intelligence in Data Mining (ICCIDM-2018).

[34] Yusoff A.F.M., Hashim A., Muhamad N., Hamat W.N.W. (2021). Application of Fuzzy Delphi Technique to Identify the Elements for Designing and Developing the e-PBM PI-Poli Module. Asian J. Univ. Educ..

[35] Patrona F., Chatzitofis A., Zarpalas D., Daras P. (2018). Motion analysis: Action detection, recognition and evaluation based on motion capture data. Pattern Recognit..

[36] Docekal J., Rozlivek J., Matas J., Hoffmann M. (2022). Human keypoint detection for close proximity human-robot interaction. arXiv.

[37] Minatour Y., Bonakdari H., Aliakbarkhani Z.S. (2016). Extension of Fuzzy Delphi AHP Based on Interval-Valued Fuzzy Sets and its Application in Water Resource Rating Problems. Water Resour. Manag..

[38] Coffey L., Claudio D. (2021). In defense of group fuzzy AHP: A comparison of group fuzzy AHP and group AHP with confidence intervals. Expert Syst. Appl..

[39] Albahri O.S., Zaidan A.A., Albahri A.S., Zaidan B.B., Abdulkareem K.H., Al-qaysi Z.T., Alamoodi A.H., Aleesa A.M., Chyad M.A., Alesa R.M. (2020). Systematic review of artificial intelligence techniques in the detection and classification of COVID-19 medical images in terms of evaluation and benchmarking: Taxonomy analysis, challenges, future solutions and methodological aspects. J. Infect. Public Health.

[40] Hassouneh A., Mutawa A.M., Murugappan M. (2020). Development of a Real-Time Emotion Recognition System Using Facial Expressions and EEG based on machine learning and deep neural network methods. Inform. Med. Unlocked.

[41] Bazarevsky V., Grishchenko I., Raveendran K., Zhu T., Zhang F., Grundmann M. (2020). BlazePose: On-device Real-time Body Pose tracking. arXiv.

[42] Bazarevsky V., Kartynnik Y., Vakunov A., Raveendran K., Grundmann M. (2019). Blazeface: Sub-millisecond neural face detection on mobile gpus. arXiv.

[43] Zhang X., Sugano Y., Fritz M., Bulling A. (2019). MPIIGaze: Real-World Dataset and Deep Appearance-Based Gaze Estimation. IEEE Trans. Pattern Anal. Mach. Intell..

[44] Mora K.A.F., Monay F., Odobez J.M. EYEDIAP: A database for the development and evaluation of gaze estimation algorithms from RGB and RGB-D cameras. Proceedings of the Eye Tracking Research and Applications Symposium (ETRA).

[45] Gu J., Yang X., De Mello S., Kautz J. Dynamic facial analysis: From Bayesian filtering to recurrent neural network. Proceedings of the 30th IEEE Conference on Computer Vision and Pattern Recognition, CVPR.

[46] Savchenko A. V Video-based frame-level facial analysis of affective behavior on mobile devices using EfficientNets. Proceedings of the IEEE/CVF Conference on Computer Vision and Pattern Recognition.

[47] McLaren L., Koutsombogera M., Vogel C. A Heuristic Method for Automatic Gaze Detection in Constrained Multi-Modal Dialogue Corpora. Proceedings of the 2020 11th IEEE International Conference on Cognitive Infocommunications (CogInfoCom).

